# X-CNV: genome-wide prediction of the pathogenicity of copy number variations

**DOI:** 10.1186/s13073-021-00945-4

**Published:** 2021-08-18

**Authors:** Li Zhang, Jingru Shi, Jian Ouyang, Riquan Zhang, Yiran Tao, Dongsheng Yuan, Chengkai Lv, Ruiyuan Wang, Baitang Ning, Ruth Roberts, Weida Tong, Zhichao Liu, Tieliu Shi

**Affiliations:** 1 Center for Bioinformatics and Computational Biology, and the Institute of Biomedical Sciences, School of Life Sciences, East China Normal University, Shanghai, 200241 China; 2 School of Statistics, Key Laboratory of Advanced Theory and Application in Statistics and Data Science-MOE, East China Normal University, Shanghai, 200062 China; 3 National Center for Toxicological Research, Food and Drug Administration, Jefferson, AR 72079 USA; 4 ApconiX Ltd, Alderley Park, Alderley Edge, SK10 4TG UK; 5 University of Birmingham, Edgbaston, Birmingham, B15 2TT UK; 6 Beijing Advanced Innovation Center for Big Data-Based Precision Medicine, Beihang University & Capital Medical University, Beijing, 100083 China

**Keywords:** XGBoost, Copy number variation, Pathogenicity, Next-generation sequencing, Machine learning

## Abstract

**Background:**

Gene copy number variations (CNVs) contribute to genetic diversity and disease prevalence across populations. Substantial efforts have been made to decipher the relationship between CNVs and pathogenesis but with limited success.

**Results:**

We have developed a novel computational framework X-CNV (www.unimd.org/XCNV), to predict the pathogenicity of CNVs by integrating more than 30 informative features such as allele frequency (AF), CNV length, CNV type, and some deleterious scores. Notably, over 14 million CNVs across various ethnic groups, covering nearly 93% of the human genome, were unified to calculate the AF. X-CNV, which yielded area under curve (AUC) values of 0.96 and 0.94 in training and validation sets, was demonstrated to outperform other available tools in terms of CNV pathogenicity prediction. A meta-voting prediction (MVP) score was developed to quantitively measure the pathogenic effect, which is based on the probabilistic value generated from the XGBoost algorithm. The proposed MVP score demonstrated a high discriminative power in determining pathogenetic CNVs for inherited traits/diseases in different ethnic groups.

**Conclusions:**

The ability of the X-CNV framework to quantitatively prioritize functional, deleterious, and disease-causing CNV on a genome-wide basis outperformed current CNV-annotation tools and will have broad utility in population genetics, disease-association studies, and diagnostic screening.

**Supplementary Information:**

The online version contains supplementary material available at 10.1186/s13073-021-00945-4.

## Background

Gene copy number variants (CNVs) are a type of structural variant (> 50 bp), characterized as duplications or deletions of genomic segments in specific DNA regions [1]. For humans, CNVs are more prevalent than single nucleotide variants (SNVs) in terms of base-pair length. On average, each individual carries approximately 1000 CNVs. On aggregate, CNVs cover ∼ 4 million bp across the genome [2]. CNVs are believed to originate via diverse mutational mechanisms such as errors in replication, meiotic recombination, and repair of double-strand breaks [2]. Evidence has mounted that CNVs make a significant contribution to rare variants involved in rare diseases [3–6] and more common diseases such as cancers [7, 8] and neurodevelopmental disorders [9–11].

Rapid advancements in emerging genomics technologies provide unprecedented breadth and depth to detect single nucleotide variations [12–14] and complex structural variants such as CNVs [15–17]. Furthermore, global collaborations established by large consortium efforts have enhanced our understanding of the distribution and functionality of CNVs across different ethnic groups [3, 18]. Consequently, a growing number of CNVs have been identified and curated in genetic variant repositories [19–21]. Concurrent with the technical advances in CNV identification, unraveling CNV pathogenicity remains a significant challenge. Computational approaches offer great opportunities to the scientific and clinical communities to predict the phenotypic impact of CNVs.

Approaches for predicting CNV pathogenicity can be divided into three types. In the first approach, aggregation of per-base single nucleotide polymorphism (SNP) pathogenicity scores within CNV intervals are used to determine the pathogenic effect of CNVs. One of the examples is SVscore [22], which calculates the pathogenic impact for CNVs by combining the SNP pathogenicity scores [23]. In the second approach, rule-based strategies prioritize CNVs, related to their pathogenic effects. The American College of Medical Genetics and Genomics (ACMG) and the Clinical Genome Resource (ClinGen) jointly proposed a guideline for the interpretation and reporting of constitutional CNVs [24]. The guideline suggested scoring metrics based on reported cases, consistency of phenotype, the pattern of inheritance, and the pathogenic mechanisms of variants to rate the CNV pathogenicity for clinical utility. However, the implementation of the guideline depends heavily on individual opinions. Clinical and genetics expertise is required, limiting its application for large-scale DNA sequencing data [25]. In the third approach, gene-based haploinsufficiency predictions are used to estimate the effect of CNVs. In this, gene dosage sensitivity is a significant determinant of the pathogenicity of genetic variants. Several attempts to estimate the impact of CNVs based on haploinsufficiency have been made [26–28]. For example, Huang et al. [27] developed a linear discriminant classifier to predict gene-based haploinsufficiency. The model integrated genomic, evolutionary, functional, and protein-protein interaction network-related features. A haploinsufficiency score was proposed to discriminate between pathogenic and benign CNV deletions, with the aim of highlighting pathogenic CNVs that were more likely to be clinically relevant. However, these features employed in the developed models mainly focused on protein-coding regions, overlooking the intergenic regions. Moreover, no single available approach considers the distribution of CNVs across ethnic groups to more precisely predict likely CNV pathogenicity.

**Fig. 1:**
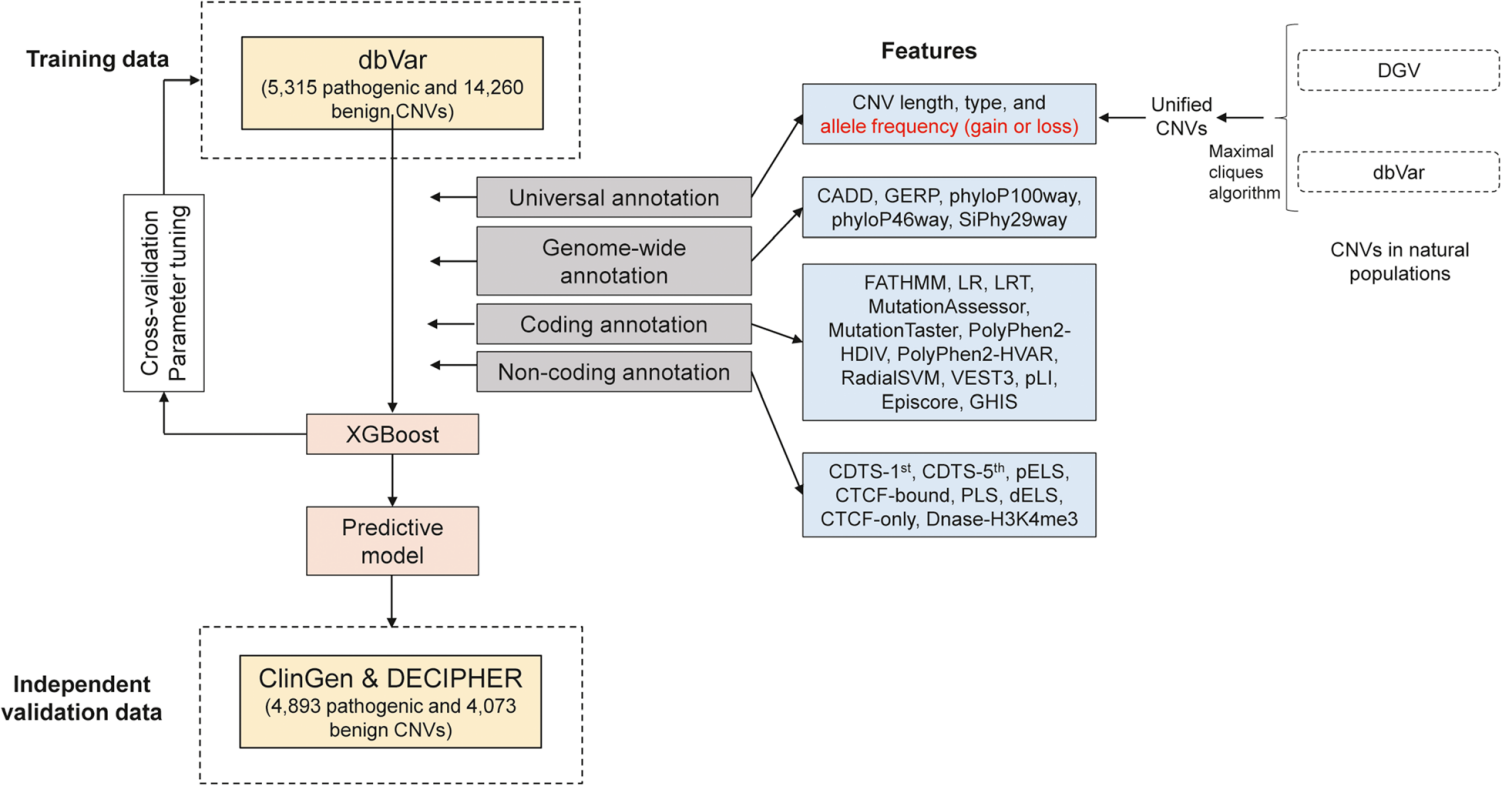
Workflow of X-CNV model training and validation. The model was trained based on the XGBoost algorithm using 30 predictive features of 5315 pathogenic and 14,260 benign CNVs from dbVar and was validated in 4893 pathogenic and 4073 benign CNVs from ClinGen and DECIPHER. The features were categorized into four types, including universal annotation, genome-wide annotation, coding annotation, and non-coding annotation. The allele frequency (AF) of CNVs was calculated based on the unified CNVs from DGV and dbVar

Sequencing Quality Control Phase II (SEQC-II), led by the U.S. FDA, is the most current initiative to develop actionable best practices for sequencing data analysis and to facilitate the clinical implementation of genomics technologies [29]. As part of the FDA-led SEQC II effort, here we introduce a novel computational framework X-CNV for CNV pathogenicity prediction (www.unimd.org/XCNV). X-CNV encompasses four major components: (1) CNV data curation and normalization, (2) model construction, (3) model evaluation, and (4) model interpretation and application (Fig. 1). X-CNV incorporates the most comprehensive CNV data and annotations by integrating diverse publicly available genetic variant repositories. To boost prediction power, informative features such as genomics, genome region, variation types, and population genetics were incorporated. More importantly, a meta-voting prediction (MVP) score was proposed to measure quantitively the CNV pathogenic effect. In contrast to any previous similar prediction models, we trained X-CNV on CNVs from both gene and intergenic regions. The utility of X-CNV was demonstrated using rare diseases, cancer predisposition, and population genetics.

## Implementation

### Data curation

To curate benchmark CNV data for development of X-CNV, we reprocessed high-quality CNV data from multiple sources including dbVar [30] (https://www.ncbi.nlm.nih.gov/dbvar/), ClinGen [20] (https://clinicalgenome.org/), DECIPHER v10.1 [31] (https://decipher.sanger.ac.uk/), and Database of Genomic Variants [19] (DGV, http://dgv.tcag.ca/dgv/app/home). The coordinates of the CNV regions were recalculated and unified based on GRCh37/hg19 by using the UCSC genome browser liftOver tool (https://genome.ucsc.edu/cgi-bin/hgLiftOver). Specifically, we collected 14,076,147 CNVs from DGV and dbVar. After removing the CNVs with the same coordinates from dbVar and DGV, we obtained 11,788,451 CNVs from 87,935 samples, which were used for CNV unification. The population information of those samples was collected from dbVar and DGV and summarized into nine ethnic groups (Additional file 1: Table S1): African/African American (AFR, *n* = 1284), Latino/Admixed American (AMR, *n* = 889), Ashkenazi Jewish (ASJ, *n* = 147), East Asian (EAS, *n* = 2114), Finnish (FIN, *n* = 103), Non-Finnish European (NFE, *n* = 11,122), South Asian (SAS, *n* = 4537), other (OTH, *n* = 390), and unknown (UKN, *n* = 67,349). Furthermore, CNVs shorter than 10 MB were used for model development and validation, and those longer than 10 MB were excluded since they were extremely likely to be pathogenic. Ultimately, 5315 pathogenic and 14,260 benign CNVs in dbVar were retained for model development (Additional file 1: Table S2), and 4893 pathogenic and 4073 benign CNVs curated from ClinGen and DECIPHER were used for model validation (Additional file 1: Table S3).

### CNV unification

To merge CNVs that were potentially identical but were from different platforms and bioinformatics pipelines, we developed a novel strategy to identify CNVs with close coordinates based on a maximal-clique algorithm. Firstly, we divided CNVs into groups based on chromosomal location. Within each chromosome, the distance between any two CNVs was calculated as below:

1 $$ {D}_{i,j}=\max \left(\left|{S}_i-{S}_j\right|,|{E}_i-{E}_j|\right) $$

where *S*_*i*_ and *E*_*i*_ are the start and end positions of the *i*th CNV, and *D*_*i*, *j*_ denotes the distance between CNV_*i*_ and CNV_*j*_.

We defined 100 bp as a genomic window (GW), representing the lower limit distance that can distinguish between two CNVs. Given a 100 bp GW, the distance *D*_*i,j*_ between CNV_*i*_ and CNV_*j*_ was converted to a binary label:

2 $$ {\mathrm{Similarity}}_{i,j}=\left\{\begin{array}{cc}0& {D}_{i,j}>100 bp\\ {}1& {D}_{i,j}<100 bp\end{array}\right. $$

**Fig. 2:**
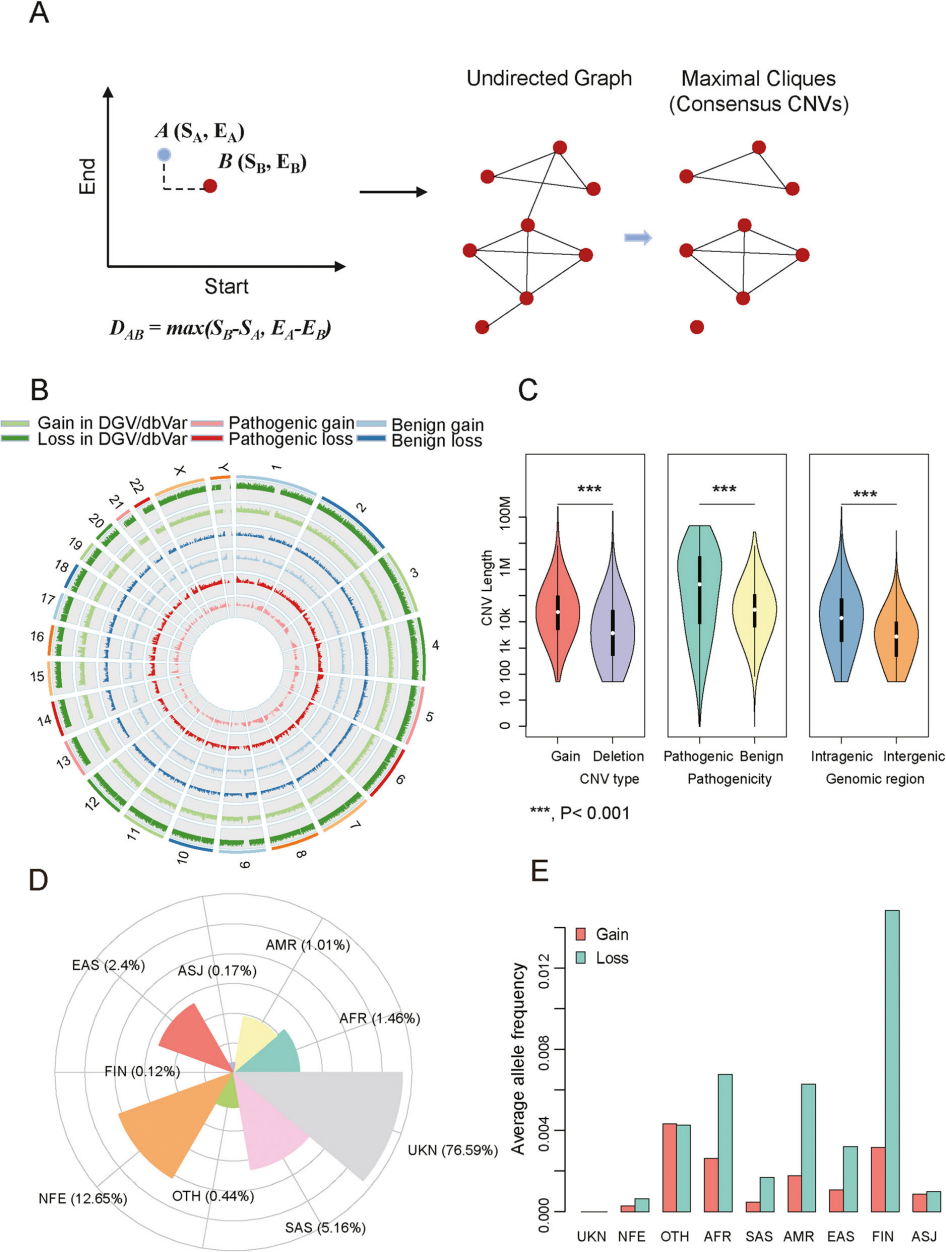
Strategy to unify potentially identical CNVs and the general properties of the unified CNVs in a natural population. **A** Schematic diagram depicting the use of maximal clique algorithm to unify CNVs. **B** Coverage of unified CNVs on the human genome. **C** The different lengths between gain and loss, pathogenic and benign, intragenic, and intergenic CNVs. **D** Proportions of the samples in the subpopulations from DbVar. **E** Population allele frequency (PAF) of gain and loss in the subpopulations

We then constructed an undirected CNV network by connecting CNVs if their binary similarity label equals one. It should be noted that the CNV network consisted of one or more subnetworks. The challenge in looking for identical CNVs was to identify the cliques in a given undirected CNV subnetwork by an iterative method (Fig. 2A). Specifically, for each loop, the maximal clique was determined from the subnetwork. The remaining nodes and edges by excluding the nodes within this maximal clique were used to construct an updated subnetwork for the next loop. If CNVs within a subnetwork are fully connected (i.e., cliques), it is indicated they are identical. In this study, the maximal cliques were identified by R igraph package with *graph_from_adjacency_matrix* and *maximal.cliques* functions, respectively.

### Feature calculation

The X-CNV used four categories of features, including universal, coding region, noncoding, and genome-wide features that were selected automatically during model development (see Additional file 1: Table S4).

The universal annotation consisted of CNV length, CNV type (gain or loss), and population-based allele frequency (AF) for each CNV. The CNVs in the natural population were collected from DGV [19] and dbVar [30] databases. The population-based AF of each CNV was calculated by comparing it to the curated CNVs in the natural populations. If the queried CNV shared at least 70% reciprocal overlap in size and location with the curated CNV, we used the population-based AF information of the curated CNV to represent that of the queried one. We employed reciprocal overlap (RO) identify the common CNV regions. The RO cutoffs ranging from 50% to 70% were used by previous studies [32–35]. In this paper, we used the stringent empirical value, 70%, as the threshold, in order to maximumly eliminate the false positives. Otherwise, we assigned zero for the queried CNV as its population-based AF for model development.

The coding annotation included various deleteriousness prediction scores from dbNSFP version 2.6 [36], a database of functional predictions and annotations for human missense and splicing SNVs (http://sites.google.com/site/jpopgen/dbNSFP). The prediction scores consist of Functional Analysis Through Hidden Markov Models (FATHMM) [37], logistic regression (LR) [38], likelihood ratio test (LRT) [39], MutationAssessor [40], MutationTaster [41], Polymorphism Phenotyping-2 (PolyPhen2) [42], Radial Kernel Support Vector Machine (RadialSVM) [38], Sort Intolerant from Tolerant substitutions (SIFT) [43], and Variant Effect Scoring Tool (VEST3) [44]. Furthermore, scores for evaluating the haploinsufficiency, including probability of being loss-of-function intolerant (pLI) score [28], Episcore, and GHIS (genome-wide haploinsufficiency score) were also employed and downloaded from ftp://ftp.broadinstitute.org/pub/ExAC_release or the supplementary materials of the publications [45, 46]. It is worth noting that LR score was calculated using the logistic regression based on nine deleteriousness prediction scores (SIFT [43], PolyPhen-2 [42], GERP++ [47], MutationTaster [41], Mutation Assessor [40], FATHMM [37], LRT [37], SiPhy [48] and PhyloP [49]), and the maximal minor allele frequency (MMAF) observed in diverse populations of the 1000 Genomes project [50]. All the functional deleteriousness scores were built based on machine learning algorithms such as hidden Markov models (HMM), logistic regression, random forest, and support vector machine (SVM). The functional deleteriousness scores were downloaded using the ljb26_all hg19 version of ANNOVAR [51]. Since the deleteriousness prediction scores were calculated at locus-level, we then calculated these scores for CNVs by dividing the sum of the scores of the variants falling within the CNV regions by the covered CNV length.

The non-coding features contained Context-Dependent Tolerance Scores (CDTS, http://www.hli-opendata.com/noncoding/) [52] and candidate *cis*-regulatory elements (cCREs) [53] including promoter-like sequence (PLS), proximal enhancer-like sequence (pELS), distal enhancer-like (dELS), CTCF-bound, CTCF-only, and DNase-H3K4me3. CDTS calculates the absolute difference of the observed variation from the expected variation, representing the likelihood of a base mutation in the human genome. The PLS was defined as sequences falling with 200 bp (center to center) of an annotated GENCODE transcription start site (TSS) and having high DNase and H3K4me3 signals. The pELS and dELS referred to genomic regions with high DNase and H3K27ac and a low H3K4me3 signals proximal (200–2000 bp) and distal (> 2000 bp) to TSS. The CTCF binding sites with high DNase and CTCF signals and those with low H3K4me3 and H3K27ac signals were defined as CTCF-bound and CTCF-only, respectively. DNase-H3K4me3 cCREs have high H3K4me3 signals but low H3K27ac signals and do not fall within 200 bp of a TSS. The CDTSs locating lower than 1% or 5% percentile were used as the CDTS scores for CNVs. The cCREs that mapped to hg38 were collected from SCREEN (Search Candidate cis-Regulatory Elements by ENCODE, https://screen.encodeproject.org/) database, and then converted to an hg19 version by UCSC genome browser liftOver tool (https://genome.ucsc.edu/cgi-bin/hgLiftOver). The scores of *cis*-regulatory elements were calculated by dividing CNV length by the length of the relevant regulatory regions.

Genome-wide annotation includes the scores of Combined Annotation Dependent Depletion (CADD) [54], GERP [47], phyloP_100way [49], phyloP_46way [49], and SiPhy-29way [48]. CADD integrates diverse genome annotations and scores any possible human single nucleotide variant (SNV) or small insertion/deletion (indel) event. The GERP [47], phyloP_100way [49], phyloP_46way [49], and SiPhy-29way [48] were conservation scores calculated by multiple alignments of vertebrate species and measurements of evolutionary conservation using Genomic Evolutionary Rate Profiling (GERP) [47], phyloP [49], and SiPhy [48] algorithms, respectively. These genome-wide annotation scores were also downloaded by using the ANNOVAR with ljb26_all annotation. The genome-wide features were calculated using the sum of the base-wise scores within the CNVs divided by the covered CNV length. For CNVs without overlapped regions with SNVs, we imputed the minimal values of calculatable CNVs for model development.

### X-CNV model development

CNVs from dbVar were used for X-CNV model development (Additional file 1: Table S2), while those from DECIPHER and ClinGen were used for model validation (Additional file 1: Table S3). To evaluate model performance without information leaking, we excluded CNVs of dbVar sharing at least 50% reciprocal overlap with ClinGen or DECIPHER. The CNV intersection was measured by using the bedtools with intersect operation. Ultimately, 5315 pathogenic and 14,260 benign CNVs in dbVar were retained for model development.

The X-CNV was developed using the XGBoost, a gradient boosting tree model, based on four different categories of features. The booster and learning algorithms were two vital parameters in XGBoost and were determined by 100-time 10-fold cross validations. Three boosters including gblinear, gbtree, and DART and learning algorithms, including regression with squared loss (reg:squarederror), regression with squared log loss (reg:squaredlogerror), logistic regression (reg:logistic), and logistic regression for binary classification (binary:logistic), were considered for parameter selection. The models with the highest median of the area under a curve (AUC) values were selected as the optimized model. Receiver operation characteristic (ROC) analysis was used to calculate the AUC value in an R ROCR package. The classifier, feature importance, and cross-validation were implemented in a R xgboost package with *xgboost*, *xgb.importance*, and *xgb.cv* function, respectively [55].

### X-CNV model validation

The developed X-CNV model was validated using the CNVs from ClinGen and DECIPHER (Additional file 1: Table S3). As the CNV length and CNV type were two important universal features in the X-CNV model, we evaluated the X-CNV performance in CNVs with different CNV lengths and types. Specifically, the CNVs in the validation set were divided into four groups based on the quartiles of CNV length. Furthermore, the performance of the X-CNV models was assessed by gain or loss CNV types.

We used the probabilistic values yielded from the XGBoost model as the meta-voting prediction (MVP) scores to quantitatively measure CNV pathogenicity. Furthermore, we employed a ROC-AUC analysis to determine the cut-offs for MVP scores that could discriminate benign, likely benign, uncertain, likely pathogenic, and pathogenic CNVs by minimizing the absolute difference between sensitivity and specificity with the R cutpointr package [56].

To compare the X-CNV model to the state-of-the-art approaches for CNV pathogenicity prediction, we used three methodologies, including SVscore [22], AnnotSV [57], and ClassifyCNV [58]. Specifically, for SVscore, the CNVs in ClinGen and DECIPHER were first converted to the VCF (Variant Call Format) file. Subsequently, the CNVs were annotated by refGene gene annotation. The sum, max, and mean of the CADD scores in the left, right, and span breakends and the beginning of the left and right breakends to the end of the truncated transcript of the CNVs were calculated for those CNVs. For AnnotSV [57] and ClassifyCNV [58], both methodologies provided a score to categorize the CNV pathogenicity into five classes, i.e., pathogenic, likely pathogenic, variant of uncertain significance (VUS), likely benign, and benign, based on ACMG classification guidelines to assess CNV pathogenicity [24]. Here, we considered the CNVs predicted to be pathogenic and likely pathogenic as positives and the ones predicted to be likely benign and benign as negatives. Subsequently, we used six other performance metrics, including MCC, accuracy, F1 score, Fowlkes–Mallows index, sensitivity, and specificity for the model comparison, as shown in the following formulas,

3 $$ \mathrm{MCC}=\frac{TP\ast TN- FP\ast FN}{\sqrt{\left( TP+ FP\right)\ast \left( TP+ FN\right)\ast \left( TN+ FP\right)\ast \left( TN+ FN\right)}} $$

4 $$ accuracy=\frac{TP+ TN}{TP+ TN+ FN+ FP} $$

5 $$ F1\ \mathrm{score}=\frac{2 TP}{2 TP+ FP+ FN} $$

6 $$ \mathrm{Fowlkes}-\mathrm{Mallows}\ \mathrm{index}=\sqrt{\frac{TP}{TP+ FP}\bullet \frac{TP}{TP+ FN}} $$

7 $$ \mathrm{sensitivity}=\frac{TP}{TP+ FN} $$

8 $$ \mathrm{specificity}=\frac{TN}{TN+ FP} $$

### X-CNV application

To investigate the performance of X-CNV on the rare disease and cancer predisposition gene (CPG), we employed the 1666 pathogenic CNVs with a definite phenotype from DECIPHER database were used for (Additional file 1: Tables S5 and S6). To identify whether pathogenic CNVs-related phenotype is rare disease-specific, we employed Human Phenotype Ontology [59] (HPO), which could be downloaded from Ontobee [60] (http://www.ontobee.org/). The HPO constructed a “Human Phenotype Hierarchy Structure” (HP-HS) with 14 layers based on the hierarchical relationship between the phenotypes (ontology terms). Notably, the term “phenotypic abnormality” is used as ancestors of all the terms in the HP-HS. The second layer of HP-HS contains the information of disease categories. Subsequently, the pathogenic CNV-related phenotypes (HPO terms) from the DECIPHER database v10.1 were mapped onto the second layer of the HP-HS to extract rare disease-related phenotypes.

The cancer predisposition genes (CPGs) used were collected from a previous study [61], which curated 58 CPGs shared between two studies [62, 63]. The pathogenic gains harboring oncogenic CPGs and losses harboring tumor-suppressing CPGs in DECIPHER database were used to calculate their MVP scores by the X-CNV model. The 4893 pathogenic and 4073 benign CNVs curated from ClinGen and DECIPHER (the validation dataset) were used for the case study about population genetics (Additional file 1: Table S3). We first calculated the MVP scores for those CNVs and assigned them one of the five pathological categories based on the optimized probabilistic cut-off value. The allele frequency within a specified ethnic group was calculated as the percentage of samples carrying the CNV within this ethnic group.

### Webserver construction

We utilized the Apache HTTP server as a web server, developed by PHP (Version: 7.0.12, https://www.php.net/) programming. Data interaction was implemented by HTML5, JavaScript, jQuery. All data in XCNV are stored and managed in MySQL database (Version: 5.7.17, https://www.mysql.com/). Data analyses were mainly carried out by the R (Version 3.6.0, https://www.r-project.org/) or python (Version 3.7.6, https://www.python.org/) script.

## Results

### Genome-wide benchmarking of CNVs

The breakpoint resolutions of the same CNVs vary in different genomics technology platforms and variation calling pipelines. To eliminate the discordance of the breakpoints among potentially identical variants, we developed a strategy to unify virtually identical CNVs with different breakpoint resolutions based on the maximal clique algorithm [64] (Fig. 2A, see the “Implementation” section). Over 14 million CNVs curated in dbVar [30] and DGV [19] were unified using this strategy. Consequently, we obtained a total of 557,892 unified CNVs for the analysis. To investigate the coverage of the unified CNVs across the human reference genome, we mapped these unified CNVs onto different chromosomes. The unified CNVs covered over 93.7% of the autosomal chromosomes (except chromosome Y), suggesting CNVs were prevalent genetic variants across the human reference genome (Fig. 2B and Additional file 1: Table S7). Notably, chromosome 17 was covered by the most CNVs (99.76%), while chromosome Y had the lowest coverage of CNVs (49.16%). One possible reason for this low coverage of CNV could be the euchromatic regions of human chromosome Y, which are not transcribed in healthy populations [65]. Furthermore, the average length of CNVs was greater in 1. gain versus loss, 2. pathogenic versus benign and 3. intragenic versus intergenic comparisons (Fig. 2C).

It is well-recognized that the allele frequency of genetic variants underpins phenotypic diversity [66–69]. We assigned 87,935 samples with population information from the dbVar [30] and DGV [19] to nine ethnic groups (Additional file 1: Table S1): African/African American (AFR, *n* = 1284), Latino/Admixed American (AMR, *n* = 889), Ashkenazi Jewish (ASJ, *n* = 147), East Asian (EAS, *n* = 2114), Finnish (FIN, *n* = 103), Non-Finnish European (NFE, *n* = 11,122), South Asian (SAS, *n* = 4537), other (OTH, *n* = 390), and unknown (UKN, *n* = 67,349). Particularly, the number of CNVs in NFE, SAS, EAS, AFR, and AMR subpopulations occupied over 1% of the total CNVs (Fig. 2D). We further calculated the allele frequency of the CNVs in each ethnic group. The average allele frequency in deletions was consistently higher than that in gains across the nine ethnic groups (Fig. 2E). The larger CNVs are more likely to be gene-disruptive. Our observation that gains have a larger size than deletions in the natural population may indicate that larger deletions are subjected to stronger purifying selection than larger gains been removed from the population. Therefore, the deletions that evolve under neutral evolutionary pressure tend to be small to medium-sized and have higher allele frequencies than the gains. A similar observation was also reported by Itsara et al. [70].

### Model construction

The pathogenic annotations of CNVs were assigned to pathogenic, likely pathogenic, uncertain, likely benign, and benign categories. Since the effect categories likely pathogenic, uncertain, likely benign are often ambiguous, we only employed CNVs annotated as pathogenic and benign for model development. We utilized the 5315 pathogenic and 14,260 benign CNVs from dbVar [30] as a training set (Additional file 1: Table S2). X-CNV uses a total of 30 predictive features that are divided into four types: universal-based features (4), coding region-based features (13), noncoding region features (8), and genome-wide-based features (5). The features could also be categorized as variant- and gene-level terms. The detailed feature information and their categories were listed in Additional file 1: Table S4.

**Fig. 3:**
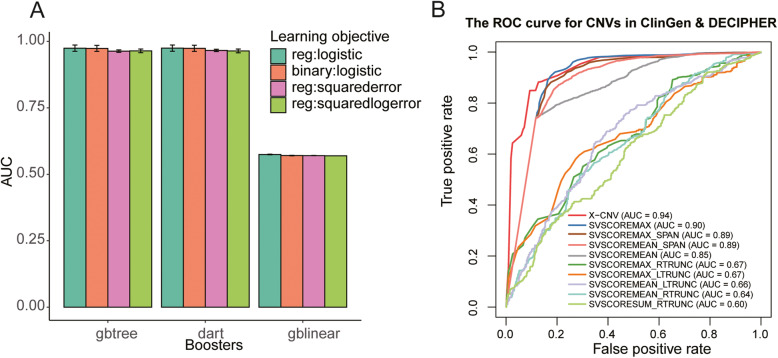
Performance and important features of X-CNV models. **A** Distribution of AUC value in the models during parameter tuning by 100-time tenfold cross-validation. **B** ROC curves for X-CNV and SVScores in the validation set (ClinGen & DECIPHER)

X-CNV uses an XGBoost classifier to distinguish pathogenic from benign CNVs. The hyperparameters of XGBoost were optimized using 100-time 10-fold cross validations. Consequently, the XGBoost model with the gbtree booster and logistic regression yielded the highest AUC (0.9740 ± 0.0058), indicating the best performance (Fig. 3A). We used the optimized hyperparameters to develop the XGBoost model with the whole training set, yielding an AUC value of 0.96. Furthermore, we used an independent validation set to further verify the developed XGBoost model, which consisted of 4893 pathogenic and 4073 benign CNVs curated from ClinGen and DECIPHER [31] (Additional file 1: Table S3).

To further compare the developed X-CNV and other the-state-of-art structural variation pathogenicity perdition approaches, we employed the SVScore [22], AnnotSV [57], and ClassifyCNV [58]. The highest AUC values (0.94) were achieved by developed X-CNV, showing an improvement of 3.5% ~ 33.7% compared to the SVScores [22] (Fig. 3B). X-CNV outperformed the AnnotSV and ClassifyCNV in five of six performance metrics, i.e., MCC, accuracy, Fowlkes–Mallows index, F1 score, and specificity, except sensitivity across all CNVs, CNV gain, and loss (Table 1). For example, X-CNV achieve a MCC of 0.65, improving 140.7% and 912.5% over AnnotSV and ClassifyCNV, respectively. The AnnotSV yielded the highest sensitivity (i.e., overall 0.96, gain 0.92, and loss: 0.98). However, the lowest specificity (i.e., overall 0.21, gain 0.13, and loss 0.35) indicated that the AnnotSV tends to predict the pathogenicity of queried CNV pathogenic or like pathogenic. The X-CNV provided the most balanced sensitivity and specificity, demonstrating its superior ability to distinguish false positives and negatives.

**Table 1:** Model performance of X-CNV, AnnotSV, and ClassifyCNV on the independent validation set

Metrics	X-CNV			AnnotSV			ClassifyCNV		
	All	Gain	Loss	All	Gain	Loss	All	Gain	Loss
MCC	**0.65**	**0.41**	**0.70**	0.27	0.06	0.46	− 0.08	− 0.24	0.01
Accuracy	**0.83**	**0.75**	**0.88**	0.62	0.38	0.79	0.48	0.34	0.57
F1 score	**0.84**	**0.58**	**0.92**	0.73	0.49	0.88	0.56	0.32	0.68
Fowlkes–Mallows index	**0.84**	**0.58**	**0.92**	0.76	0.56	0.87	0.56	0.34	0.68
Sensitivity	0.85	0.52	0.96	**0.96**	**0.92**	**0.98**	0.62	0.47	0.68
Specificity	**0.8**	**0.86**	**0.70**	0.21	0.13	0.35	0.31	0.28	0.34

### Important features of the X-CNV

**Fig. 4.**
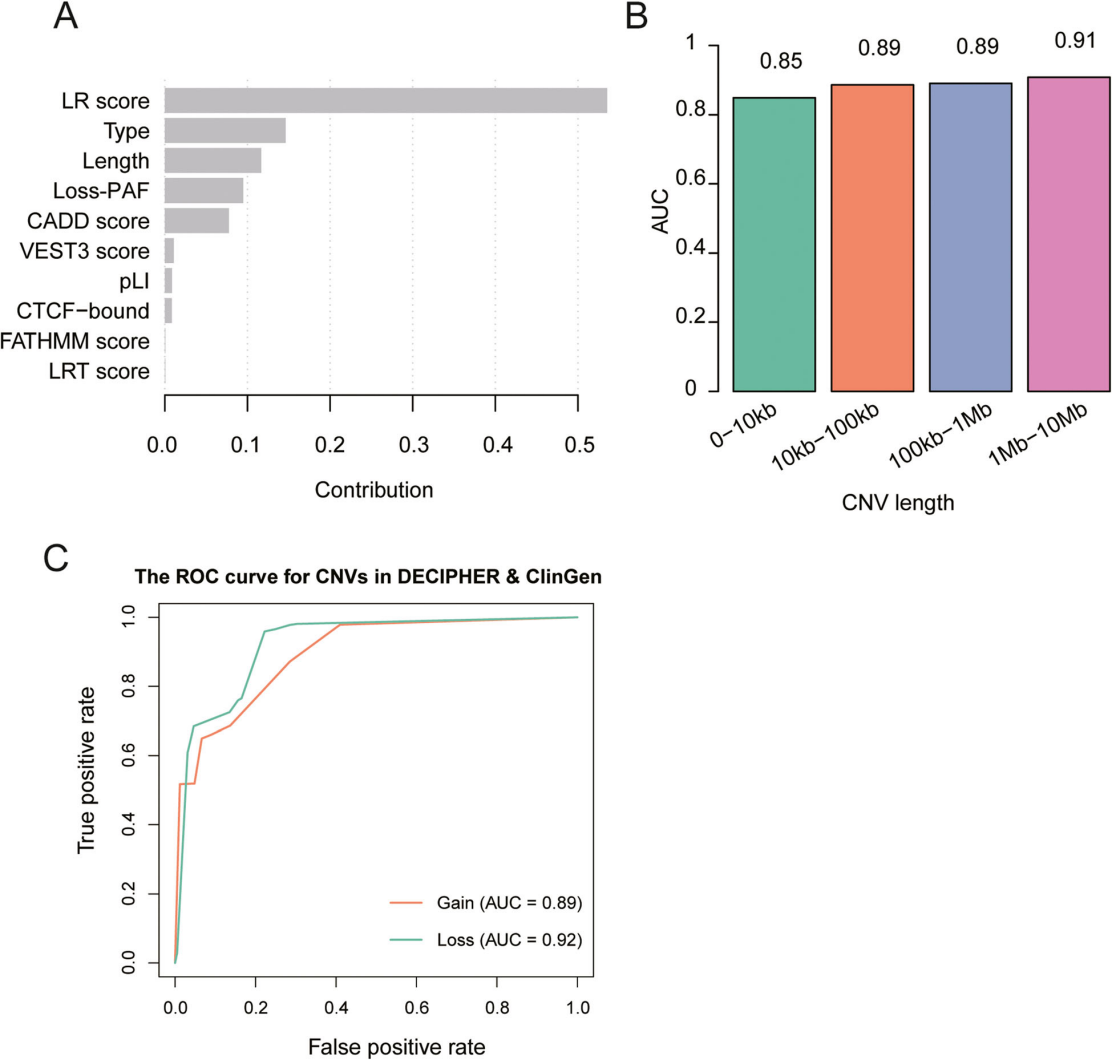
The important features of X-CNV model for CNV pathogenicity prediction. **A** The contribution of the ten important features for CNV pathogenicity prediction. **B** AUC values of the X-CNV model for CNVs with four different CNV lengths. **C** Receiver operating characteristic (ROC) curves for CNV gain (orange) and loss (green), respectively

To enhance the X-CNV model explainability, we extracted the top ten predictive features from the optimized XGBoost model (Fig. 4A). The top ten most predictive features consisted of 4 coding features (LR score, VEST3 score, FATHMM score and pLI), 3 universal features (CNV type, length, and loss-PAF), 2 noncoding features (PLS and CTCF−bound), and 1 genome-wide feature (CADD). The logistic regression (LR) score contributed to over 50% of the performance of the XGBoost model. Since the LR score integrates diverse information from nine deleteriousness prediction scores, it is not surprising that the LR score substantially contributes to the model performance. CNV length and type were also two prominent universal features of X-CNV model performance.

We further examined X-CNV model performance in CNVs with different lengths and CNV types. The CNVs in the validation set were divided into four groups 0~10 kb, 10 kb ~ 100 kb, 100 kb ~ 1 Mb, and 1 Mb ~ 10 Mb. The AUCs were over 0.85 for all four groups indicating that the X-CNV model could achieve high performance in CNVs with different lengths, although longer CNV length tended to predict more precisely (Fig. 4B). X-CNV achieved AUCs of 0.85 and 0.89 for both small and medium-sized CNVs (i.e., 0~10 kb, 10 kb ~ 100 kb), indicating that the X-CNV model could achieve a good performance on small to medium-sized CNVs. Furthermore, the X-CNV model yielded a higher AUC for CNV losses (0.92) compared with CNV gains (0.89) (Fig. 4C).

### MVP score for quantitatively measuring CNV pathogenicity

**Fig. 5:**
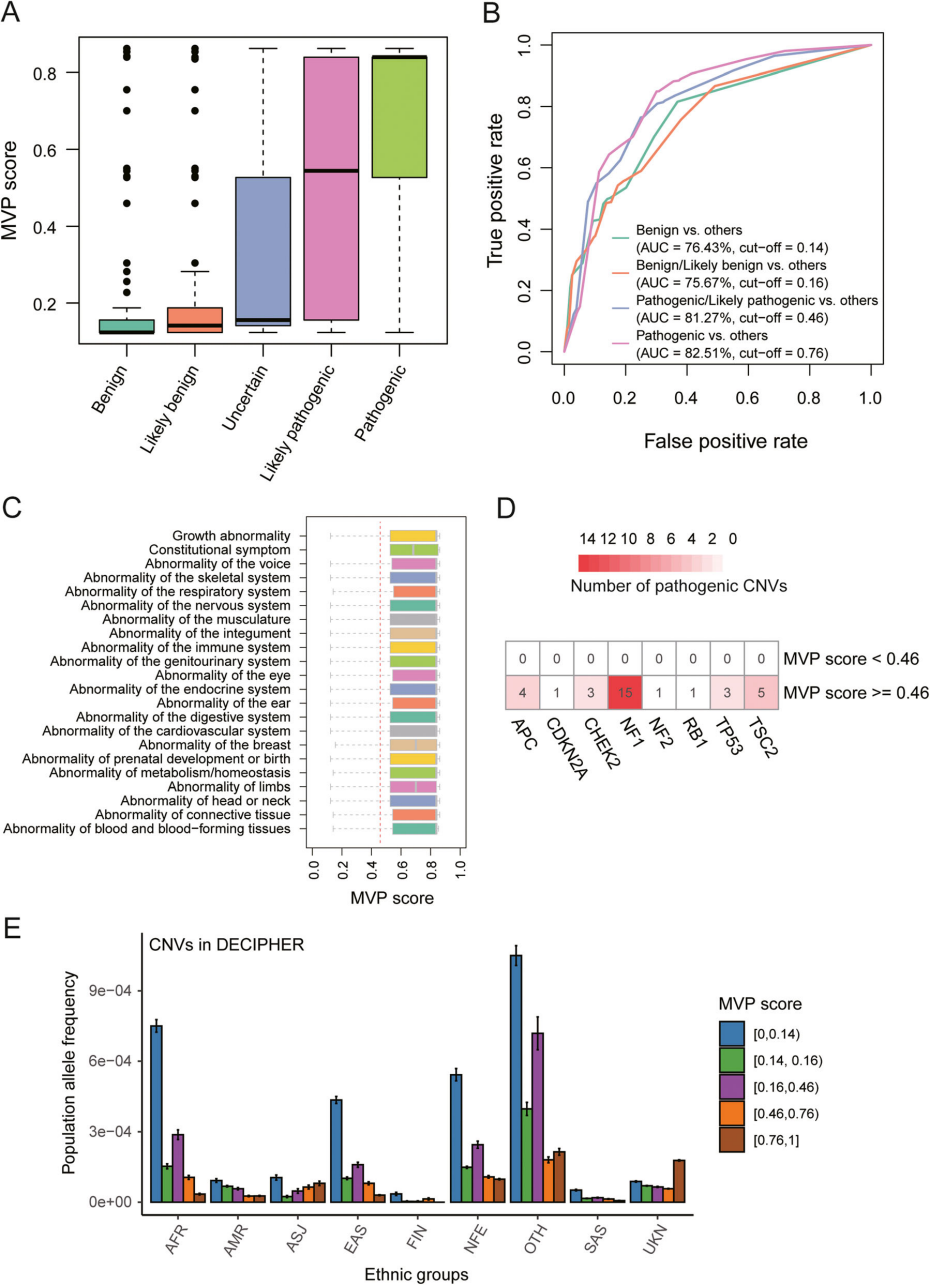
The separating capability of the meta-voting prediction (MVP) score in the pathological categories and its application to rare disease, hereditary tumor, and population genetics. **A** Distribution of MVP scores in the five pathological categories. The points above the boxes represent the outliers. **B** AUC values and cutoffs of the meta-voting prediction (MVP) scores to separate the five pathological categories. **C** The distribution of MVP scores in the pathogenic CNVs of 22 rare disease types. **D** The number of CNVs harboring cancer predisposition genes and being predicted as pathogenic or likely pathogenic (MVP > 0.46). **E** The allele frequency distribution of the CNVs categorized by the MVP scores. The average and 95% confidence intervals of population allele frequency of the CNVs categorized by the MVP scores within the ethnic groups

To quantitatively measure the relationship between CNV and pathogenicity to support potential clinical applications, we developed a meta-voting prediction (MVP) score based on probabilistic values generated from XGBoost algorithms (see the “Implementation” section). Specifically, we applied X-CNV to a total of 31,942 CNVs with pathologic effect annotations from ClinGen and DECIPHER (Additional file 1: Table S3). The probabilistic values of X-CNV could distinguish the different pathologic effects with statistical significance (Fig. 5A). We further employed ROC-AUC analysis to define the optimized probabilistic cutoff value for each pathological category. We found high AUC values in all the pathological categories with optimized probabilistic cutoff values (Fig. 5B). Consequently, MVP scores were more than 0.76, between 0.46 and 0.76, between 0.16 and 0.46, between 0.14 and 0.16, and below 0.14 for pathogenic, likely pathogenic, uncertain, likely benign, and benign, respectively.

### Case study 1: rare diseases

Rare and recurrent CNVs have been associated with various types of rare diseases [3]. We investigated how the developed X-CNV may be useful in distinguishing CNV pathogenicity in different types of rare diseases. Specifically, the 1666 CNVs with a definite phenotype from DECIPHER database were categorized into 22 normalized rare disease types based on Human Phenotype Ontology (HPO) [71]. The X-CNV predicted 1408 of 1666 CNVs (84.51%) as pathogenic/likely pathogenic. The 25% quantiles of MVP scores for all the rare disease types were more than 0.46, suggesting CNVs are likely to be predicted as pathogenic or likely pathogenic by X-CNV model (Fig. 5C and Additional file 1: Table S5). Of 22 rare disease types, more than 85% CNVs of 19 categories are predicted pathogenic or likely pathogenic, indicating the pathogenicity of CNVs could be highly distinguished in these rare disease types. About 17% of CNVs (4/23) in one rare disease type (Abnormality of the breast) were uncertain (MVP score: 0.16 ~ 0.46). We further check the 4 CNV based on ACMG guidance lines. As a result, 3 of the 4 CNVs are considered pathogenic, and one CNV is deemed to be uncertain based on the ACMG guideline, suggesting further clinical evidence is needed to verify the pathogenicity of these CNVs.

### Case study 2: cancer predisposition genes (CPGs)

Germline mutations in cancer predisposition genes (CPG) confer high or moderate increased risks of cancer [72]. We next considered how the X-CNV could identify the pathogenic CNVs located in CPGs. We curated 32 CNV losses with a definite phenotype from DECIPHER (Additional file 1: Table S6), located in 8 tumor-suppressing CPGs, including *APC*, *CDKN2A*, *CHEK2*, *NF1*, *NF2*, *RB1, TP53*, and *TSC2* [61]. Notably, all these CNVs were predicted as pathogenic or likely pathogenic (MVP score > 0.46, Fig. 5D). Furthermore, we associated the CPGs harboring CNVs with the phenotypes annotated in DECIPHER (Additional file 2: Fig. S1). We observed that some of these phenotypes, such as adenomatous colonic polyposis, cystic renal dysplasia, and hemangioma, were also associated with malignant tumors as reported in previous studies [73–75].

### Case study 3: population genetics

Studies of CNV in healthy populations provide a basis for comparison when studying the types of CNVs that are most likely to be pathogenic and are more likely to have no appreciable clinical effect tailored to a specific population. To address this, we further examine the power of our proposed X-CNV to differentiate the CNV pathogenicity in the nine ethnic groups as mentioned above. We observed that predicted pathogenic CNVs showed much lower frequencies than the predicted benign CNVs from the validation set (Fig. 5E and Additional file 1: Table S3, the average frequency of pathogenic CNVs vs. benign CNVs, *P* value < 0.05), which is consistent with the epigenetic finding that the pathogenic variants were extremely rare in healthy populations due to purifying selection [76].

## Discussion

X-CNV has a unique ability to integrate diverse human genome information towards a quantitative measure of CNV pathogenicity on the whole genome-scale. X-CNV created a curated benchmark CNV list by combining publicly available CNV resources to generate the most comprehensive list of feature-related CNV pathogenicity. The X-CNV yielded an outstanding performance and provided a “one-stop” solution for CNV pathogenicity estimation. To assist with clinical application, the meta-voting prediction (MVP) scores based on probabilistic values generated from XGBoost algorithms could distinguish pathogenic/likely pathogenic, uncertain, and benign/likely benign CNVs with AUC values of only 72.9% and 81.83%. MVP scores were successfully applied to rare diseases and inheritable cancers. In population genetics, the pathogenic CNVs showed much lower frequencies than benign CNVs, suggesting that pathogenic CNVs were rarely prevalent in a healthy population due to purifying selection.

It is worth considering additional investigations to further improve the model performance and confirm the findings from this study: (1) comparing X-CNV and the other three state-of-the-art CNV pathogenicity prediction approaches (i.e., SVscore, AnnotSV, and ClassifyCNV) were based on only one independent validation set. We highly recommended further evaluation with more accumulated annotated CNV pathogenicity in the future. It may be a good solution to investigate the combined power of these CNV pathogenicity approaches for an enhanced prediction power. (2) Considering the limited annotated CNV pathogenicity data in the noncoding regions, we could not perform a comprehensive assessment of X-CNV performance on the CNV located in the non-coding regions. It may explain the reason that noncoding features contributed very little to the X-CNV model. (3) In the current version of X-CNV, we employed 30 different genome/gene and variant-related features. The predictive power of the X-CNV model may be improved by adding some noncoding features like EIGEN [77] and LINSIGHT [78] at variant-level and coding features at gene-level such as RVIS [79], Multinet [80], MSC [81], and GDI [82]. (4) We employed the XGBoost algorithm to develop the X-CNV model. Further investigations on other machine learning, especially deep learning algorithms, may improve performance.

To facilitate the real-world application of our proposed X-CNV model, we developed a user-friendly web server to encourage submissions from users. Following ACMG (American College of Medical Genetics) guidelines [24], predicted CNVs were classified into five categories based on the proposed MVP score, including most likely pathogenic, likely pathogenic, uncertain, likely benign, and most likely benign. Furthermore, we characterized CNVs by integrating various database resources and curated information using text mining techniques, such as pathogenicity annotations assigned by CNV-related databases, clinical evidence, CNV-associated clinical phenotypes, and allele frequencies in different ethnic groups and experimental data from knockout mouse models. The comprehensive characterization enables users to associate CNVs with specific phenotypes and other underlying mechanisms.

Another key feature is that the X-CNV model can be updated as new data and knowledge on CNVs emerge, serving as a complementary tool for prioritizing CNV pathogenicity in a clinical setting. As emerging genomic technologies for accurately detecting CNVs and clinical evidence on pathogenicity of CNVs accumulate, we envisage that X-CNV will become a valuable tool in connecting complex genetic traits with a disease, offering a positive impact for promoting public health.

## Conclusions

In summary, X-CNV can quantitatively prioritize functional, deleterious, and disease-causing CNV on a genome-wide basis and has broad utility in population genetics, disease-association studies, and diagnostic screening.

## Availability and requirements

Project name: X-CNV

Project home page: www.unimd.org/XCNV (web server) or https://github.com/kbvstmd/XCNV.

Operating system(s): Linux

Programming language: Shell, R 3.6

Other requirements: None.

License: GNU license - GPL 2.0 (GNU General Public License. version 2) (https://opensource.org/licenses/GPL-2.0).

Any restrictions to use by non-academics: none.

## Supplementary Information

**Additional file 1.** Table S1. The sample information of the natural population collected from dbVar and DGV. Table S2. The 19,575 CNVs from dbVar used for model training. Table S3. The 8966 CNVs used for model validation and 22,976 likely pathogenic, likely benign, or uncertain CNVs used to quantitively measure the pathogenic effect from ClinGen and DECIPHER. Table S4. The 30 predictive features used by XGBoost algorithm. Table S5. The 1666 CNVs with a definite phenotype from DECIPHER database. Table S6. 32 CNV losses with a definite phenotype from DECIPHER, which were located in 8 tumor-suppressing cancer predisposition genes (CPGs), including APC, CDKN2A, CHEK2, NF1, NF2, RB1, TP53, and TSC2. Table S7. The coverage of the unified CNVs on the chromosomes of the human genome.

**Additional file 2.** Figure S1. The cancer predisposition genes (CPGs) located within the pathogenic CNVs and their corresponding phenotypes.

## Data Availability

The following public data resources and tools were listed: dbVar (https://www.ncbi.nlm.nih.gov/dbvar/) [30] ClinGen (https://clinicalgenome.org/) [20] DECIPHER (v10.1, https://decipher.sanger.ac.uk/) [31] Database of Genomic Variants (DGV, http://dgv.tcag.ca/dgv/app/home) [19] dbNSFP http://sites.google.com/site/jpopgen/dbNSFP [36] pLI ftp://ftp.broadinstitute.org/pub/ExAC_release [28] Episcore and GHIS: The supplementary materials of the publications [45, 46] CDTS http://www.hli-opendata.com/noncoding/ [52] SCREEN (Search Candidate cis-Regulatory Elements by ENCODE, https://screen.encodeproject.org/) [53] PHP (v7.0.12, https://www.php.net/) HTML5 (v1, https://www.w3.org/TR/html5/) JavaScript (v1.8, https://developer.mozilla.org/) jQuery (v3.1.1, https://jquery.com/) MySQL database (v5.7.17, https://www.mysql.com/). R (v3.6.0, https://www.r-project.org/) python (v3.7.6, https://www.python.org/) R xgboost package (v1.1.1.1, https://cran.r-project.org/web/packages/xgboost/index.html) R igraph package (v1.2.6, https://cran.r-project.org/web/packages/igraph/index.html) Human Phenotype Ontology (HPO) (https://hpo.jax.org/app/) [59] Ontobee (http://www.ontobee.org/) [60] UCSC genome browser liftOver tool (https://genome.ucsc.edu/cgi-bin/hgLiftOver).

## Abbreviations

CNV: Copy number variation
MVP: Meta-voting prediction
SNV: Single nucleotide variant
ACMG: American College of Medical Genetics and Genomics
ClinGen: Clinical Genome Resource
AF: Allele frequency
RO: Reciprocal overlap
FATHMM: Functional Analysis Through Hidden Markov Models
LR: Logistic regression
LRT: Likelihood ratio test
MMAF: Maximal minor allele frequency
SVM: Support vector machine
HMM: Hidden Markov models
CDTS: Context-Dependent Tolerance Scores
CADD: Combined Annotation Dependent Depletion
ROC: Receiver operation characteristic
HPO: Human Phenotype Ontology
