# Supplementary material for: X-CNV: genome-wide prediction of the pathogenicity of copy number variations
Source: Genome Med. 2021 Aug 18;13:132. doi: 10.1186/s13073-021-00945-4 (PMC8375180; doi:10.1186/s13073-021-00945-4)

Figure S1. The cancer predisposition genes (CPGs) located within the pathogenic CNVs and their corresponding phenotypes

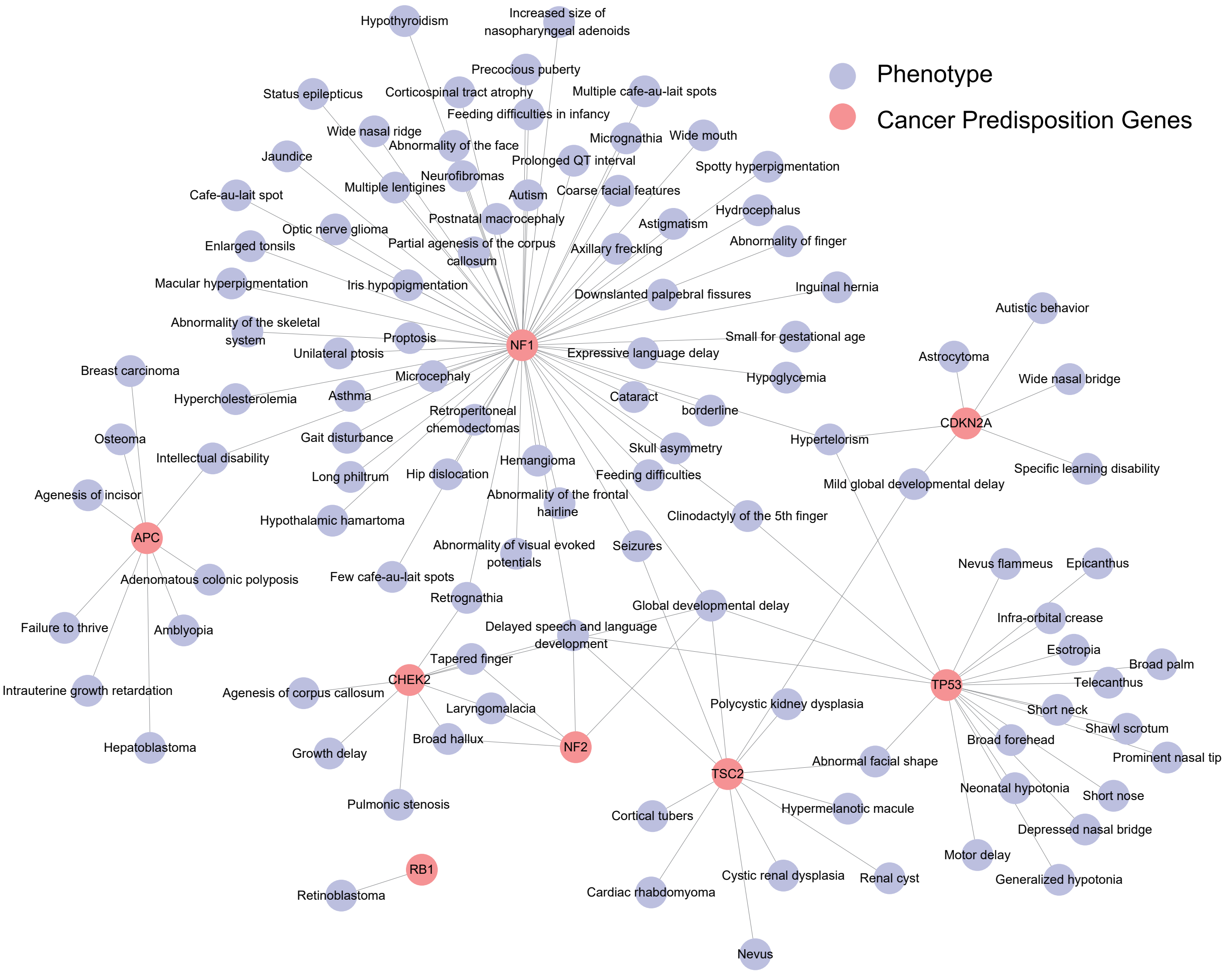

Supplement: Supplementary file 2 — Additional file 2. Figure S1. The cancer predisposition genes (CPGs) located within the pathogenic CNVs and their corresponding phenotypes. [file 13073_2021_945_MOESM2_ESM.pdf]
